# Niche Variation in Endemic *Lilium pomponium* on a Wide Altitudinal Gradient in the Maritime Alps

**DOI:** 10.3390/plants11060833

**Published:** 2022-03-21

**Authors:** Ninon Fontaine, Perrine Gauthier, Gabriele Casazza, John D. Thompson

**Affiliations:** 1 Centre d’Ecologie Fonctionnelle et Evolutive (CEFE), University of Montpellier, CNRS, EPHE, IRD, 34293 Montpellier, France; perrine.gauthier@cefe.cnrs.fr (P.G.); john.thompson@cefe.cnrs.fr (J.D.T.); 2 Dipartimento di Scienze della Terra dell’Ambiente e della Vita (DISTAV), University of Genoa, Corso Europa 26, 16132 Genoa, Italy; gabriele.casazza@unige.it

**Keywords:** ecological micro-niche, altitude, distribution range, centre-periphery hypothesis, endemic plant

## Abstract

The relationship between altitudinal and ecological gradients has long been a dominant theme in plant ecology; moreover, how species respond to climate change has renewed this interest. Mediterranean mountains are often hotspots of endemism, and some endemic species have local distributions that span different climatic belts; hence, local variations in topography and fine-scaled niche conditions may play crucial roles in their persistence along such gradients. Studies of the fine-scaled niche are, however, very rare; most studies involve broad-scale variations in climatic parameters. The Turban lily, *Lilium pomponium* L. is endemic to the Maritime and Ligurian Alps, where it occurs across a wide altitudinal gradient. Previous work has shown no link between climatic marginality and geographic range limits on morphological traits and genetic variability; however, possible variations of local topographic and ecological parameters have not yet been examined. The objective of this paper is to characterise local ecological niche conditions of *L. pomponium* populations in the different bioclimatic zones it occupies along the altitudinal gradient. The species occurs in four main types of microecological niches. One of these niche types, with a high mineral cover, is the most abundant—type 2: it was detected in 39% of sampled quadrats and occurs across the whole bioclimatic gradient. Other niche types are more limited to subsections of the gradient: type 3 (in 19% of sampled quadrats) is restricted to high-altitude sites (>1070 m.a.s.l.) and is characterised by high vegetation and litter cover; type 4 (26%) corresponds to more forested habitats on substrates with low water retention capacities, in more inland zones close to the centre of *L. pomponium* distribution and across a range of altitudes; and type 1 (16% of quadrat) only occurs in the Mediterranean part of the gradient, close to distribution limits in pockets of soil among large blocks of rocks, mainly found at mid-altitudes. Despite heterogeneity in the spatial locations of niche types, there is no correspondence between ecological gradients and the distribution limits of this species. Knowledge of the fine-scaled ecological conditions that determine niche types is thus essential for conservation management of the habitats of this species and for the exploration of its possible response to ongoing climate change.

## 1. Introduction

Ecological gradients in relation to altitude have long been a dominant theme in plant ecology. As a result, we now benefit from a wealth of studies detailing patterns in the proportion of endemic species and species diversity [1,2,3,4], associations among species [5], pollination strategies [6,7,8], reproductive traits [9,10,11], and potential variation in functional traits [12,13,14] along altitudinal gradients. In more recent years, the observation of changes in species distribution and their colonisation of higher latitudes and altitudes in relation to warming climates [15,16,17], have renewed interest in the understanding of the ecological interactions that govern distribution patterns in mountain plants. In addition, the observation of rapid genetically based adaptation to relaxed selection pressures on micro-environmental gradients that incur milder winters [18] indicates how changes in distribution patterns may be linked to localised topographic and ecological niche conditions that interact with climate change.

Mediterranean mountains have attracted much attention in this respect. The comparison of conjoint variation in temperature and drought with elevation has allowed vegetation communities to be classified into seven different bioclimatic elevation belts [19,20,21], three of which are representative of truly Mediterranean conditions, and four others represent transitional environments towards mountain and alpine vegetations. Along these gradients, species diversity and the proportion of endemic plants in the local flora show striking patterns, and some endemic species occur on wide altitudinal gradients, a distribution pattern that may be intimately linked to historical periods of climate change [2]. What may be crucial here is how such species show variation in their fine-scaled multi-dimensional ecological niche in different climatic belts and broad habitat types. Indeed, several recent studies illustrate the importance of quantifying multiple dimensions of the ecological niche (species composition and richness of functional types, the proportion of mineral elements and other biotic cover variables, and soil chemistry) on a highly localised scale, for our understanding of differences in species range sizes and limits, and the presence in different broad habitat types [22,23,24,25].

**Figure 1:**
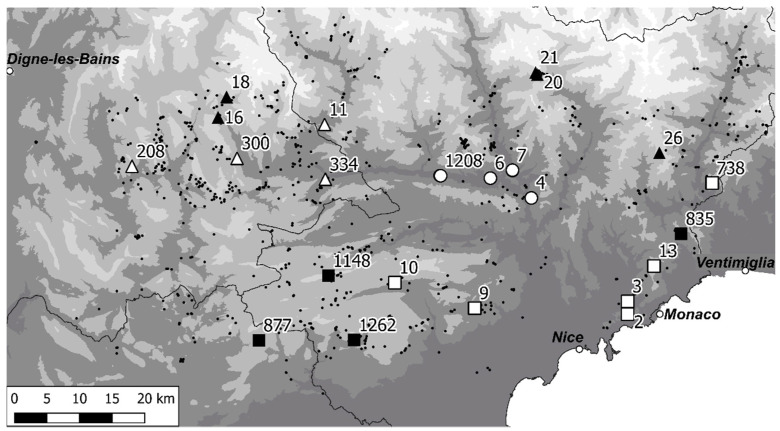
Location of the sampled sites (large symbols) in the distribution of *Lilium pomponium* (small black circles). Open circles—low-altitude stations, open squares—mid-altitude Mediterranean, open triangles—mid-altitude temperate mountain, black squares—high-altitude Mediterranean, black triangles—high-altitude temperate mountain. Black lines are the limits of French departments.

The Turban lily, *Lilium pomponium* L. is endemic to the Maritime and Ligurian Alps (Figure 1) and occupies a wide altitudinal gradient [26]. There is no apparent link between gradients of climatic conditions and trait variation other than a shift towards smaller flowers at high elevation [27]. In a study of genetic variability, Casazza et al. [28] found no decline in genetic variability in peripheral populations of this species relative to those in the centre of its distribution, as would be predicted by the so-called centre–periphery hypothesis [29]. Climatic and/or ecological conditions may in fact have a mosaic distribution in the area of presence of *L. pomponium*, where mixed alpine and Mediterranean influences may interact along localised topographic gradients at different distances from the Mediterranean Sea. Hence, there is a clear need for a detailed study of localised ecological niche conditions across the range of altitude and different bio-climatic zones where this species is present, in order to understand the causes of its presence in different belts.

The objective of this study was to examine whether the characteristics of the local scale ecological niche of endemic *Lilium pomponium* have a specific distribution in relation to either (or both) an altitudinal gradient or a spatial, geographic gradient from the centre to the periphery of the species’ distribution. The aim here was to test whether fine-scaled ecological niche parameters (topographic, edaphic and biotic parameters) vary towards the limits of these two macroecological gradients. To do so we examined three main issues. First, we tested whether the species showed variations in its topographic niche in relation to altitudinal variations that may have indicated its precise relation to climatic parameters along this gradient. Second, we explored variations in fine-scaled ecological niche parameters in a stratified sample of populations along the altitudinal gradient in order to test for variations that facilitated the presence of this species along the gradient. Third, we examined the hypothesis that populations show variations in their ecological niches at the limits of the distribution of this species, both in terms of an ecological gradient of altitude and spatial geographic range limits. To assess any variability in performance along the altitudinal and geographic gradients, we also quantified phenotypic traits and local abundance.

## 2. Results

### 2.1. Local Topography

**Figure 2:**
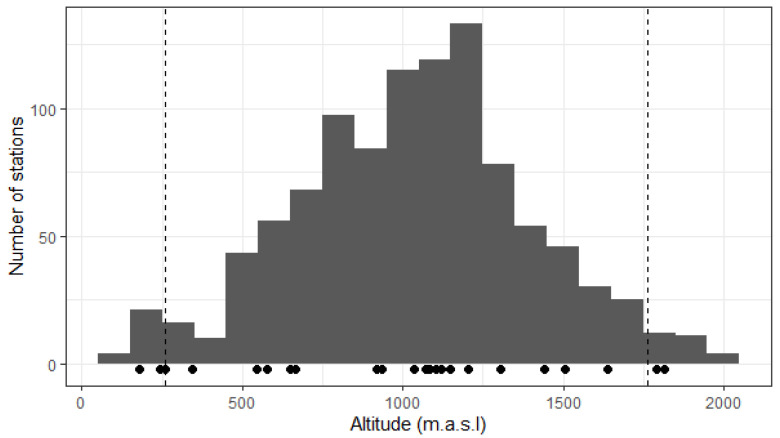
Altitudinal distribution of all known French locations of *Lilium pomponium*. Binwidth is 100 m. Dashed lines represent quantiles at the 2.5% and 97.5% levels: 95.0% of stations are between 260 and 1762 m. Black dots under the histogram correspond to the locations of the 23 sampled populations.

**Figure 3:**
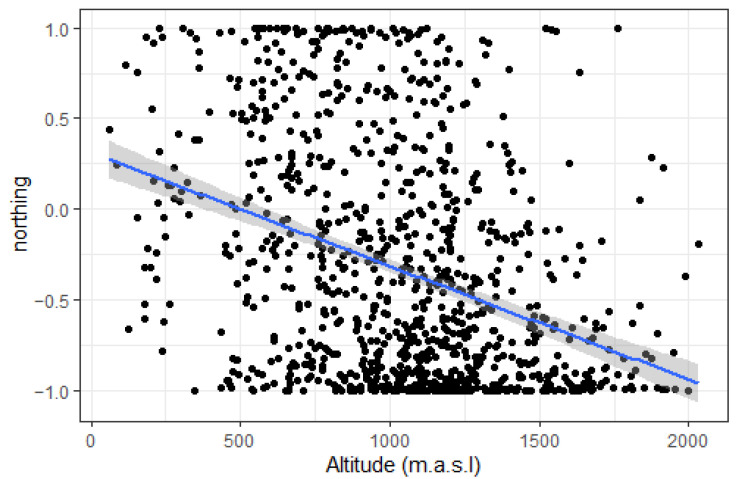
Linear relationship between northing and altitude for all known French locations of *Lilium pomponium* (black dots). The blue line corresponds to the linear regression (*p*-value < 0.001, adjusted-R^2^ = 0.126) and the grey band is the confidence interval at the 0.95 level.

*Lilium pomponium* occurs across a wide altitudinal gradient from 61 to 2033 m.a.s.l. with a major part of known locations (90%) between 455 and 1651 m (Figure 2). Northing is the only topographic parameter that significantly changes along the altitudinal gradient (*p*-value < 0.001, adjusted-R^2^ = 0.126). Although slopes vary from 1° to 80°, there is no significant linear relationship between altitude and easting (*p*-value = 0.879), nor between altitude and topographic position index (*TPI*) (*p*-value = 0.180). The relationship between altitude and slope is significant but weak (*p*-value = 0.007 but adjusted-R^2^ = 0.006). Sites of *L. pomponium* present in mid-altitudes are exposed in all directions whereas high-altitude locations are less exposed to the north and low-altitude sites are less exposed to the south (Figure 3).

### 2.2. Micro-Niche Diversity

**Figure 4:**
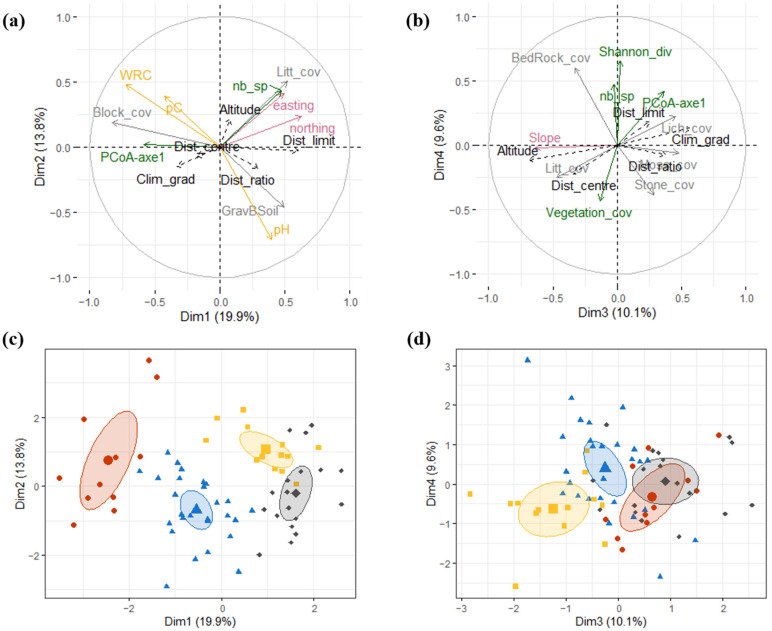
Illustrations of the four main micro-niche types revealed by MFA and HCPC analyses. (**a**,**b**) Positions of parameter variables following MFA for the four main environmental axes. Each colour represents a group of micro-niche characteristics: soil cover (grey), biotic niche (green), topographic niche (pink), edaphic niche (mustard). Only the 10 variables that best represent the plan are plotted (according to their cos^2^). “Marginality” variables are supplementary variables in the MFA and plotted in black. (**c**,**d**) quadrat positions according to the HCPC results. The four HCPC clusters are plotted with different symbols: red circles for micro-niche type 1, blue triangles for type 2, orange squares for type 3, grey diamonds for type 4. Ellipses correspond to confidence interval at the 0.99 level. The first two axes of the MFA are on the left (**a**,**c**), while axes 3 and 4 are on the right (**b**,**d**).

**Figure 5:**
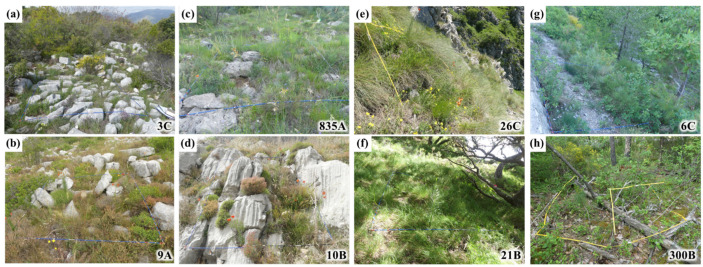
Illustrations of the four different niche types of *Lilium pomponium*: (**a**,**b**) niche type 1, (**c**,**d**) niche type 2, (**e**,**f**) niche type 3, (**g**,**h**) niche type 4. Upper line (**a**,**c**,**e**,**g**) corresponds to the typical quadrats closest to the cluster centre in the MFA, lower line (**b**,**d**,**f**,**h**) to the typical quadrats furthest from other clusters.

Four main dimensions revealed by the multiple factor analysis (MFA) describe 53.3% of the micro-niche space occupied by *L. pomponium* populations. A group of abiotic variables represent major contributions to distinguish clusters of quadrats in the first two dimensions, and a group of biotic variables differentiate quadrat clusters for dimensions 3 and 4. Four main types of micro-niche are identified by hierarchical clustering on principal components (HCPC) applied to these data (Figure 4). A narrative of their main characteristics (Table 1, Figure 5) is as follows.

In micro-niche type 1, *L. pomponium* occurs in pockets of soil with a high water retention capacity between large blocks of rocks that are stabilised in fairly flat areas with abundant low woody shrubs (*Thymus vulgaris*, *Euphorbia spinosa*) and low species diversity typical of Mediterranean scrublands. Eleven quadrats are assigned to this micro-niche type that is almost exclusively present (10 quadrats) in a mid-altitude zone (from 543 to 934 m.a.s.l.).

Micro-niche type 2 is characterised by its mineral nature with a high cover of bedrock and stones. This type of local niche is the most abundant and widely distributed with quadrats that occur across the whole altitudinal gradient, in both Mediterranean zones (to the south) and more temperate mountain zones (north).

Micro-niche type 3 is characterised by high vegetation and litter cover with two abundant grass species, *Sesleria caerulea* and *Helictotrichon sempervirens*, which are typical of high-altitude communities [30]. All quadrats that correspond to this type of niche occur at higher altitudes (>1070 m.a.s.l.) in both temperate mountain and Mediterranean zones.

Micro-niche type 4 corresponds to more forested habitats on substrates with variable amounts of gravel, moss, and bare soil with a low water retention capacity. This type of niche does not occur in the two (mid- and high-altitude) Mediterranean bio-climate zones, all 18 quadrats occur in the three more continental, inland bioclimatic zones (low altitude, and mid- and high-altitude temperate mountain zones).

### 2.3. Marginality and Distribution Limits

**Figure 6:**
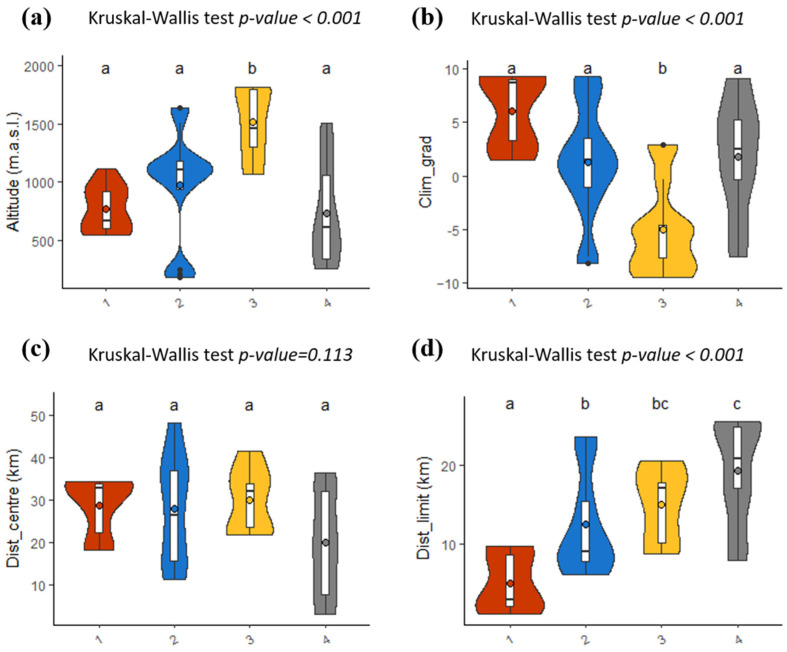
Differences in position along ecological gradients and in relation to geographic distribution limits for the four main micro-niche types of *Lilium pomponium*. Ecological position is expressed in relation to (**a**) altitude and (**b**) climatic gradients based on the main axis of principal component analysis (PCA) of monthly meteorological data). Geographic locations of sites represent Euclidian distance to either (**c**) the centre or (**d**) distribution limits. Kernel density for each micro-niche type shows quartiles (boxplot) and mean values (coloured circle). Code letters indicate that micro-niche means are significantly (*p* < 0.05) different in pair-wise Wilcoxon tests with Bonferroni adjustment.

The comparison of each of these niche types on climatic and spatial gradients revealed two main results. First, micro-niche type 3 is significantly different in terms of position along ecological gradients compared to other niche types (Figure 6a,b), due to its limitation to high-altitude sites that have a higher amount of winter precipitation and lower maximal temperatures during spring and summer. Second, the four micro-niche types show significant differences in their spatial location in relation to the limits of the species distribution (Figure 6d), a difference that is not however shown in relation to the centre of the distribution (Figure 6c), probably because of the east–west elongated shape of *L. pomponium* distribution (Figure 1). Niche type 1 is significantly closer to the limits of *L. pomponium* distribution in the north-west of the study area than the other niche types, and niche type 4 is significantly further away from the distribution limits than all other niche types (Figure 6d). Niche types 2 and 3 have a more overlapping geographic distribution that is spatially intermediate between niche types 1 and 4. It seems that micro-niche diversity is higher further from the distribution limits, where niche types 2, 3 and 4 all occur, than closer to the range limits, where only niche type 1 occurs (Figure 6d).

### 2.4. Abundance and Phenotype

There was no significant variation in the abundance and phenotype of *L. pomponium* (where it is present) in the different micro-niche types (Kruskal–Wallis test: *p*-value > 0.01; pair-wise Wilcoxon tests with Bonferroni adjustment: *p*-value > 0.05—Supplementary Material Figure S2) despite a slight trend towards fewer flowers and individuals in niche types 3 and 4 (Supplementary Material Figure S3).

### 2.5. Vegetation Structure

At the site scale, site 300 is different from all other sites due to a higher cover of woody vegetation > 5 m high, young trees < 2 m high, and woody litter. This site also had a high cover of artificial elements (access roads) (Supplementary Material Figure S4a). Other sites form two clusters that show significant differences amongst each other in the HCPC analysis. One cluster contains sites with a lower cover of artificial elements and relatively higher cover of herbaceous vegetation (sites 3, 7, 9, 10, 13, 18, 20, 21, 26, 334, 738, 835, 877, 1148, 1208, 1262), the other cluster presents more signs of human disturbance and less herbaceous vegetation cover (sites 2, 4, 6, 11, 16, 208) (*p*-value = 0.006 and *p*-value < 0.001, respectively, for Wilcoxon tests between the two groups, for herbaceous and artificial covers, respectively). The latter sites are also characterised by higher number of flowers per scape and higher mean plant height, but less dense populations. Micro-niche types 3 and 4 tend to have more woody cover than micro-niche 1, as found above (Supplementary Material Figure S4b).

## 3. Discussion

The results of this study reveal the importance of topographic and fine-scaled niche analyses for our understanding of plant responses to climate variation along altitudinal gradients and provide an empirical illustration of ecological variation on a local scale that is not necessarily linked to distribution limits. Our study aliments perspectives for the conservation management of the study species.

### 3.1. Climate Change: The Importance of Tracking the Fine-Scaled Niche

Interest in how plants respond to ongoing climatic change has renewed studies of their ecology and evolution along altitudinal gradients. Several studies have already indicated how increasing temperatures may enable the colonisation of higher latitudes and altitudes [15,16,17,31,32,33] or show genetically-based adaptations to relaxed selection pressures (milder winters) on local-environmental gradients [18]. There has, however, been surprisingly little attention paid to the multi-dimensional nature of ecological niche variation along altitudinal gradients. The transition zone between the Mediterranean and Alpine climate regions in the Maritime Alps, with sharp gradients over short distances and a spatial mosaic of local topographic situations [2,20,34,35,36], provides an ideal situation for such studies.

In this region, *Lilium pomponium* occurs over a wide altitudinal gradient (60–2000 m.a.s.l.) with 90% of known locations between 455 and 1650 m. In terms of altitude, this species is thus primarily a mid-altitude species but with non-negligible numbers of populations at low- and high-altitudes. High- and low-altitude populations may represent relict sites linked to historical climates. This distribution pattern can be observed in many species on different Mediterranean mountain ranges and probably results from repeated phases of migration in relation to climate changes during the glacial and inter-glacial periods of the Pleistocene [2]. Indeed, the Mediterranean region is known to be a micro-refuge of many plant species that often show sharp patterns of genetic differentiation in relation to climate history and local topography [37,38]. The current distribution of *L. pomponium* may thus be in a state of ongoing change.

An important result of our work is the finding that the mid-altitude sites, where *L. pomponium* is present, are exposed in all directions, whereas at a high altitude, locations are less often exposed to the north, and at low-altitude sites, rarely exposed to the south. This pattern suggests that *L. pomponium* is more selective in terms of its local topographic niche at each end of the altitudinal gradient and is rarely found on more north-facing aspects at high altitudes and more south-facing slopes at low altitudes. If this species is to respond to ongoing climate change, one would expect that its colonisation of high-altitude sites will initially favour more southerly (east and west) exposures, and then in later years to colonise sites at high elevation with a more northerly exposure with climate warming. At low altitudes, a trend in migration towards more northerly sites may already be operating, as our data suggest.

The study of fine-scaled ecological requirements revealed the presence of *L. pomponium* in four main micro-niche types, some of them with specific locations. The presence of *L. pomponium*, in particular niche types, indicates that it is fundamental to question how altitudinal gradients may interact with local ecological conditions and micro-topography to affect future dispersal and colonisation of mountain habitats, as reported by Mursal et al. [33]. These authors showed that the orchid *Platanthera chlorantha* occurs on an altitudinal gradient from 588 to 1043 (or more rarely 1300) m.a.s.l. in diverse forest types, where the most favourable conditions for *P. chlorantha* are temperate, warm, and humid climates, and mountain brownish soils. Niche availability may thus favour response to climate change. In our study, a response to climate change is likely to occur by the species tracking the common niche type 2 that is present across the gradient in altitude, and possible colonisation of the conditions of niche type 3 in high-altitude locations. Niche type 4 may be of less importance in a context of climate change given that it is not currently part of the niche of the study species in the mid- and high-altitude Mediterranean bioclimatic zones, and also the slight trend towards smaller size and abundance of *L. pomponium* individuals when they are present in this niche type. That said, type 4 is at least as common as, and perhaps more common than, sites of micro-niche type 3 (26% of quadrats for niche type 4 compared to 19% for type 3).

Our results clearly indicate that our study species, and perhaps many other species, will not simply migrate to higher altitude in response to climate change; they will track their localised ecological niche, which remains a rarely studied element of plant responses to changing climates. As Måskiven et al. [4] point out, there may be strong influences of site-specific factors on species presence, which may at least partly over-ride effects from more broad parameters such as altitudinal and environmental variation. Indeed a study of an endemic orchid on the island of La Réunion has reported that diverse local ecological parameters may be at least as important as altitude to explain species presence [39]. In accordance with these authors and others [40], site-specific variation in abiotic factors, such as mineral cover and soil chemistry, as well as local topography and plant community composition interact with more broad-scale climatic gradients to shape species distribution patterns along altitudinal gradients. Our study is in agreement with the findings of these different authors and illustrates that the availability of different niche types occupied by a species may be crucial to their response to ongoing climate change. We thus provide empirical example of one of the findings reported by Grytnes et al. [41], where the habitat preferences of individual species may potentially explain variations in range shifts towards higher elevations with climate warming.

### 3.2. Ecological and Range Limits, Two Separate Issues

Despite heterogeneity in the spatial location of the four niche types and variations among them in terms of their positions on the altitudinal gradients, there is no correspondence between ecological gradients and the distribution limits of this species. Micro-niche type 1 occurs at mid-altitude and, thus, in relation to the altitude–climate gradient, in macroecologically “central” conditions, but occurs spatially close to the geographic distribution limits of the species in the Maritime Alps. Likewise, micro-niche type 3 occurs primarily at high altitude, closer to the limits of the macroecological gradient (this niche type is also the most distant cluster from all other clusters in the multivariate analysis of microecological variables), but not closer to the distribution limits of the species. Hence, there is no evidence of a correlation between macroecological gradients and geographic range limits.

These results provide a parallel with the previous studies of genetic variability in relation to distribution limits and potential ecologically marginality [27,28]. Casazza et al. [28] insist on the probable effects of local topographic variation that blur any possible relation between genetic variation and distribution limits. They suggest that a decrease of genetic diversity along environmental, but not geographical gradients, may be due to the presence of low-quality habitats in the different parts of the range. This may affect population dynamics and genetics irrespective of distance from the geographical centre of the range. Furthermore, Macrì et al. [27] found no direct relationship between the presence near range limits and phenotypic performance.

In contrast, we found that the diversity of niche types is dramatically less in peripheral populations compared to central populations of *L. pomponium*. In sites close to the range limits, one primary niche type predominates, whereas further from the distribution limits, three other niche types are more common. Thus, although the trend predicted for genetic diversity to decline towards range limits [29] is not apparent in this species [28], we observed here a decline in the diversity of realised niche conditions near the distribution limits. In addition, one of the niche types is rather unique to peripheral populations, where we have no evidence for poor performance. Papuga et al. [23], in a study of eleven species that had an Ibero-Provençal distribution with central populations in the Iberian Peninsula and peripheral populations in the South of France, reported consistent differences in the fine-scaled niche characteristics (but not the broad habitat type) of the two groups of populations. Despite fine-scaled ecological niche differences in peripheral populations, these authors found no evidence of sub-optimality in these populations. Indeed, the central–peripheral hypothesis (based on a concordance between geographical peripherality and ecological marginality) that environmental conditions become harsher towards the limits of a species distribution is not followed in many cases [29]. These authors, in their review of this subject, thus cast doubt on the pertinence of a main assumption of the centre–periphery hypothesis. For genetic diversity [28] and ecological niche characteristics (this study), *Lilium pomponium* provides another example that does not support the centre–periphery hypothesis. Localised complex topography in a mosaic physical and biotic landscape may be a major element of these discrepancies.

### 3.3. Perspectives for Conservation Management

Given that migration to higher altitudes basically means colonizing conditions in a spatially reduced area relative to low altitudes [42], the conservation of high-altitude non-north-facing sites, with one or another of the different niche types detected in this study, will be important for future persistence and colonization of *L. pomponium*. This is particularly important for this species given that models based uniquely on climatic variables illustrate its relative sensitivity to future projected climate changes [43].

Slight differences in abundance and plant size illustrate that two of the niche types may represent sub-optimal niche conditions (ecological marginality) in relation to local environmental conditions. The sites in question appear to be subject to forest closures in mid- and high-altitude sites less exposed to Mediterranean climate conditions. An important issue here concerns micro-niche type 3, which is the most representative of high-altitude sites and, thus, the destination for population colonization in high elevation locations in a context of climate change. The high-altitude sites also tend to have plants that bear smaller flowers [27] that could also represent a sign of poorer viability. However, *L. pomponium* densities and the number of flowers per plant tend to be lower in this niche type, probably as a result of forest closure. Hence, colonization potential may in turn be quite low. Loss of potential habitats due to forest closures at high altitudes could reduce the possible response of this species to climate change. A more complete study of the issue of forest closure and its potential impact on the abundance of this species at multiple locations across the gradient is necessary.

In future work, field studies in the Mercantour National Park could be conducted, to assess population status and micro-niche types in the core zone of the park that has a strict regulatory conservation status relative to the non-regulated adhesion area. Such work could identify sites that may require management in terms of forest closure and localised disturbance. In addition, at the regional scale, it will be useful to evaluate the status of populations at the landscape scale, using a threat criteria that assesses the relative percentage of natural habitats or urbanisation, the fragmentation of populations, and the area of a population in a protected area, as conducted for several other species in the South of France [44].

## 4. Materials and Methods

### 4.1. Study Species and Area

*Lilium pomponium* L. (Liliaceae) is a perennial orophyte endemic to the Maritime and Ligurian Alps [26]. Over its range, *L. pomponium* is quite frequent, but often scattered and dispersed in small populations. The species grows on a calcareous substrate across a wide altitudinal range from 60 to 2000 m.a.s.l. It primarily occurs in garrigues and rocky grasslands in different types of vegetation communities, including the following phytosociological associations: *Helianthemo italici–Aphyllanthion monspeliensis*, *Lavandulo angustifoliae–Genistion cinereae*, and *Avenion sempervirentis* [26].

### 4.2. Altitudinal Variation in Topographic Parameters

Occurrences of all known populations were extracted from the SILENE database of the “conservatoire botanique national méditerranéen de porquerolles” (France) (http://www.silene.eu/index.php?cont=accueil) [45] (accessed on 3 February 2021). Only locations posterior to 1980 were considered, to avoid occurrences not collected from a GPS or potentially extinct populations. Some locations were added from previous studies on this species [27,28]. The database used here contained 1026 occurrences.

Topographic parameters were derived from a digital elevation model (DEM) (RGE Alti ^®^ version 2.0 IGN https://geoservices.ign.fr/documentation/diffusion/telechargement-donnees-libres.html) [46] (accessed on 5 September 2020). A spatial resolution of 5 m appeared as a best compromise. This scale is precise enough to study fine-scale niche variations compared to a 25 m resolution, and at the same time incorporate the lack of precision of geo-localization data for sites of presence in the SILENE database. Altitude, aspect, slope, and a standardised topographic position index (TPI) were extracted or calculated for all of the *L. pomponium* locations in the database using the “*terrain*” function in the R package *Raster* [47]. Easting and northing were calculated as *sin(aspect)* and *cos(aspect)*, respectively.

We examined the relationship between altitude and topographic parameters (slope, easting, northing, standardised topographic position index) for all known *L. pomponium* locations, with simple linear models using the R function *lm()* from the package *stats* [48].

### 4.3. Population Sampling along Altitudinal and Bioclimatic Gradients

To construct a bioclimatic zonation of the study area in relation to the altitudinal gradient, we obtained temperature and precipitation data for the twenty-five weather stations that exist across the distribution of the study species. Nineteen bioclimatic variables were computed with the R function *biovars* from the package *dismo* [49], and meteorological stations were assigned a bioclimatic position within one of five major clusters (Supplementary Material Figure S1, Table 2) using the hierarchical clustering on principal components (HCPC) function in the R package *FactoMineR* [50]. The geographical position of the twenty-five meteorological stations and their altitudes revealed two major altitudinal gradients in the study region. Both gradients have common existence in the low-altitude situation of cluster 5 (<350 m). From this cluster, one gradient includes clusters 3 (350–1100 m) and 1 (>1100 m) that occurs primarily in the more temperate northern and western parts of the study area. The second gradient includes clusters 4 (350–1100 m) and 2 (>890 m), encompassing weather stations that are almost exclusively in the southern (and eastern) parts of the study area, closer to the Mediterranean Sea. These two altitudinal trends differ primarily in relation to precipitation (Supplementary Material Table S1). There are thus two main bioclimatic gradients, one in the southern and eastern area of the species’ distribution that is mainly under a Mediterranean climate influence, and a second altitudinal gradient in the northern and western sector, under a more temperate mountain influence. We used the five clusters as a proxy for bioclimatic zones in the study area to which the known locations of *L. pomponium* were assigned: low altitude (<350 m), mid-altitude Mediterranean (350–1100 m), and high-altitude Mediterranean (>1100 m) zones in the south and east of the region, and mid-altitude temperate mountain (350–1100 m) and high-altitude temperate mountain (>1100 m) zones in the west and north of the study species’ distribution.

These groups of populations were used to stratify site selection to study the ecological niche. Five locations of *L. pomponium* in each of the five bioclimatic groups were randomly selected, favouring locations used in previous studies [27,28]. Locations highly isolated from other sites were not considered in the selection, to allow us to rapidly select a replacement site if the randomly selected site could not be sampled due to site destruction, too few individuals, or simply inaccessibility. The 25 selected locations were visited during spring 2021, from mid-May to the 30 June, depending on population phenology. Data were collected at 23 of these sites: two were abandoned due to problems of access and population size (Table 2, Figure 1).

### 4.4. Micro-Niche Characterization

Three 4 m^2^ quadrats were randomly selected in high density patches of the study species in each site. The three quadrats were at least 5 m apart in an area with a maximum radius of 20 m. Following previous studies [23], we measured the slope and exposure, and determined the identity of all species present. We then quantified the cover of ecological parameters using contact point data obtained for a grid of 100 contact points in each 4 m^2^ quadrat, i.e., one contact point every 20 cm. The following elements were assessed: bedrock, blocks (>25 cm), stones (2.5–25 cm), gravel (0.5–2.5 cm), bare soil, lichen, moss, herbaceous, and woody litter, and living plants. Each contacted plant species was identified.

A soil sample (<10 cm) was obtained for each quadrat. Soils were transferred to the laboratory in paper bags and dried at 40 °C for at least 48 h, sieved through a 2 mm grill, and stored in a cool room prior to analysis. Conductivity (conduc), expressed in milli-siemens per centimetre) and pH (pH) were measured using a Thermo Scientific Gallery discrete analyser with electrochemical units. After mixing 15 g of dry soil with 30 mL of water, we blended the solution during a 30 min period, then separated phases using a centrifuge (15 min), and measured values in the supernatant at room temperature (circa 20 °C). Water retention potential (WRP) is the percentage of water lost after drying wet soil for 48 h at 40 °C. Water retention capacity (WRC) was then calculated as the percentage of water remaining in this previously 40 °C-dried soil by a repeated drying of the sample at 105 °C for 5 h. Organic matter (OM) was estimated as the percentage of matter lost after burning a dried sample at 500 °C during 5 h. The percentages of carbon (pC) and nitrogen (pN) were determined using a Thermo Finnigan Flash EA 1112 series on 50 mg samples grinded with a crusher (3 min, frequency p 30). C:N ratio was calculated with these values.

Micro-niche characteristics were combined in a data-frame of quadrat *x* variables. Variables were structured in four groups: topographic, edaphic, abiotic/soil cover, and biotic characteristics (Supplementary Material Table S2), and analysed through multiple factor analysis (*MFA*) by using the R package *Ade4* [51]. Different types of micro-niches were defined using the clusters identified by the HCPC analysis mentioned above.

### 4.5. Ecological Gradients and Distribution Limits

Two metrics were used to assess the central/marginal positions of sites on ecological gradients: altitude and climate (combination of monthly precipitation and minimal and maximal temperature). The two parameters are correlated (correlation coefficient = −0.9), but the second is the more informative. The climatic gradient (Clim_grad) is the main axis of a principal component analysis of the monthly meteorological data, computed from the model developed by Martin, Carrega, Adnes [52], using the two metrics.

To assess niche differences in relation to possible variations between geographically central and peripheral populations of *L. pomponium*, three metrics were used: Euclidian distance to the centre of the distribution (Dist_centre), Euclidian distance to the limit of the distribution (Dist_limit), and the ratio of these two distances (Dist_ratio) [28].

### 4.6. Phenotypic Traits

To assess phenotypic variations among populations, three traits were measured on twenty individuals in each site (four individuals randomly selected in each quadrat, and eight additional individuals outside the quadrats in the same site): vegetative height (Hveg), total height (Htot), and number of flowers (Nflo). We calculated the mean and standard variations for each trait in each quadrat and site.

### 4.7. Local Abundance

At the micro-niche scale (i.e., 4 m^2^ quadrats), the local abundance of *L. pomponium* was quantified by its frequency of presence in the 100 20 × 20 cm cells of each quadrat. At the site scale, we quantified the presence/absence of individuals in forty 1 m^2^ quadrats in the immediate area of the three micro-niche quadrats. These 1 m^2^ quadrats were placed every 2 m on four 20 m long transects extending north, south, east, and west from the barycentre of the three micro-niche quadrats. The 1 m^2^ quadrats were used to assess the degree of artificialization and colonisation by woody plants. To do so, we assessed the percentage cover of each woody strata in the site by the presence/absence in the forty 1 m^2^ quadrats of artificial elements, woody litter, herbaceous vegetation, woody vegetation <2 m high (making a distinction between young trees and other woody species), woody vegetation 2–5 m high, and woody vegetation >5 m high.

All statistical analyses were conducted with R 4.1.1. [48], using packages cited in the text above.

## Figures and Tables

**Table 1:** Description of the four micro-niche types in relation to parameters analysed by HCPC on MFA, and their position along ecological (climatic) gradients and in relation to geographic distribution limits. Symbols indicate whether the mean value of the niche type is significantly higher+ or lower–than the overall mean. A sign in parenthesis indicates significantly higher standard deviation within a given micro-niche type. Quadrat number is the code for the study site and A, B, and C represent the three quadrats in a given site.

	Description of the Micro-Niche Type *	Quadrats		Percentage of Quadrats	Position along Ecological Gradients **	Geographic Limits **
**1**	+ Block cover, water retention capacity, first PCoA *** axis (characterised by *E. spinosa* and *T. vulgaris*) − Slope, Shannon diversity, litter cover, gravel and bare soil cover, number of species (+) Lichen cover, conductivity (−) pH, 4th PCoA axis (community characterised by *Stipa juncea*, *Rhamnus alaternus*, *Smilax aspera*, *Brachypodium retusum*, *Ruta angustifolia*, *Pistacia lentiscus*	2 ABC 3 ABC 9 ABC	13 C 1262 B	15.9%		Close to the limits of *Lilium* distribution
**2**	− Easting, northing (+) Bedrock cover, stone cover	4 ABC 7 ABC 10 ABC 20 ABC 208 ABC 835 ABC 877 ABC	13 AB 18 C 738 A 1262 AC	39.1%		Intermediate distance to distribution limits
**3**	+ Litter cover, vegetation cover − 1st PCoA axis (characterised by high presence of *Sesleria caerulea* and *Helictotrichon sempervirens*), easting	21 ABC 26 ABC 1148 ABC	16 A 300 A 738 BC	18.8%	High altitude	
**4**	+ Northing, easting, pH − Water retention capacity, conductivity, soil carbon content, block cover (+) Gravel and bare soil cover, moss cover	6 ABC 11 ABC 334 ABC 1208 ABC	16_BC 18 AB 300_BC	26.1%		Close to the distribution centre

* Only characteristics that are significantly different for the micro-niche type are shown. ** Only significant differences are commented. *** PCoA: principal coordinates analysis of species data.

**Table 2:** Location and characteristics of the sampled sites along the altitudinal gradient. Group codes are: BA: low altitude (<350 m), MA: mid-altitude (350–1100 m), HA: high altitude (>1100 m), med: Mediterranean, mont: subalpine.

Altitude (m)	ID	Sampling Group	Locality	Date	Habitat Type	Slope (°)	Aspect (°)
191	4	BA	Vallon d′Aïga Blanca *06450 Utelle*	19 May 2021	Garrigue, scree slope	35	250
244	7	BA	M2205 *06710 La Tour*	18 May 2021	Garrigue, scree slope	40	250
259	6	BA	D126 *06710 Massoins*	18 May 2021	Mixed woodland	35	80
336	1208	BA	les Moulières *06710 Touët-sur-Var*	15 June 2021	Shrubland	45	10
544	3	MA_med	Plateau Tercier *06340 La Trinité*	4 May 2021	Garrigue on karst	0	-
570	334	MA_mont	D610 *04320 Entrevaux*	15 June 2021	Rocky grassland	20	55
649	11	MA_mont	path to l′Aiguille *06470 Daluis*	15 June 2021	Shrubland	25	70
665	2	MA_med	Fort de la Revère *06360 Eze*	3 May 2021	Garrigue, scree slope	35	180
918	9	MA_med	Col de Vence *06140 Vence*	19 June 2021	Rocky grassland	10	150
934	13	MA_med	Col de la Madone de Gorbio *06440 Peille*	16 June 2021	Rocky grassland	35	90
1034	10	MA_med	D2 *06620 Gréolières*	19 June 2021	Garrigue on karst	45	170
1065	300	MA_mont	Verdre *04240 Annot*	14 June 2021	Forest edge	20	90
1078	738	MA_med	below Mont Tron *06540 Breil-sur-Roya*	17 June 2021	Shrubland	30	105
1099	208	MA_mont	Chemin du Jas de Bernard *04170 Moriez*	14 June 2021	Shrubland	35	180
1118	1262	HA_med	Forêt domaniale de Nans *06460 Saint-Vallier-de-Thiey*	20 June 2021	Scree slope	30	195
1142	835	HA_med	below Mont Razet *06500 Castillon*	18 June 2021	Shrubland	30	230
1205	877	HA_med	Combe de la Roque *83840 La Roque-Esclapon*	21 June 2021	Shrubland	40	235
1305	16	HA_mont	D908, ravin des Baumettes *04170 Thorame-Haute*	28 June 2021	Forest edge	45	95
1447	1148	HA_med	Col de Bleine *06750 Andon*	20 June 2021	Garrigue on karst	35	55
1505	18	HA_mont	Peyresq, ravin de la Fontaine *04170 Thorame-Haute*	28 June 2021	Shrubland	40	200
1640	20	HA_mont	l′Adrechas, la Colmiane *06420 Valdeblore*	29 June 2021	Shrubland	30	205
1791	21	HA_mont	les Aiguillettes, la Colmiane *06420 Valdeblore*	29 June 2021	Pine forest	40	95
1816	26	HA_mont	Pointe de Ventabren *06380 Moulinet*	30 June 2021	Montane grassland	45	130

## Data Availability

Not applicable.

## Supplementary Materials

The following supporting information can be downloaded at: https://www.mdpi.com/article/10.3390/plants11060833/s1. Table S1: Spatial coordinates and altitude of the 25 meteorological stations used to define bioclimatic zones for stratified sampling. Figure S1: Multivariate analysis of 19 bioclimatic variables from 25 meteorological stations (PCA–2 main axes), and clustering of the stations from HCPC. Table S2: Studied gradients and micro-niche characteristics. Figure S2: Local abundance and mean phenotype for each quadrat depending on its micro-niche type. Figure S3: Local abundance and mean number of flowers for each quadrat depending on its micro-niche type. Figure S4: Vegetation structure and artificialization level for sampled locations of *L. pomponium*, evaluated at the site scale.
