# Supplementary material for: Niche Variation in Endemic Lilium pomponium on a Wide Altitudinal Gradient in the Maritime Alps
Source: Plants (Basel). 2022 Mar 21;11(6):833. doi: 10.3390/plants11060833 (PMC8954139; doi:10.3390/plants11060833)
Supplement: Supplementary file 1 [file plants-11-00833-s001.zip › plants-1621171-supplementary.pdf]

Supplementary material

Table S1. Spatial coordinates and altitude of the 25 meteorological stations used to define bioclimatic zones for stratified sampling (minimal and maximal altitudes for each cluster are highlighted in bold type).

Bioclimatic cluster	Station ID	Longitude	Latitude	Altitude (m.a.s.l.)	Bioclimatic characteristics *
1	6005001	7.01	43.92	1173	High values for bio2, 3, 14, 17, 18: important precipitation even during summer, strong daily and annual thermic variations
	6077006	7.363	43.929	1443	
	6094002	6.931	44.1	1784	
	6102001	7.13	44.07	1130	
	6125001	6.75	44.15	1642	
2	6037002	6.92	43.75	1268	High values for bio12, 13, 16 : important precipitations in general
	6050002	7.05	43.79	985	
	6081001	6.809	43.814	1525	
	83044003	6.471	43.659	892	
3	4115001	6.62	44.03	1090	Intermediate values for bioclimatic variables, but a bit more precipitation during the warmest quarter for cluster 3
	4136001	6.52	43.98	920	
	6074005	7.31	43.97	550	
	6099004	6.89	43.96	442	
	6127006	7.26	44.07	899	
	6163007	7.59	44.04	636	
4	6038001	6.99	43.67	388	
	6059003	7.36	43.74	677	
	6075007	7.21	43.85	691	
	6091003	7.43	43.78	1106	
	6118002	6.809	43.679	694	
	6136005	7.434	43.863	843	
5	6033002	7.21	43.79	78	High values for bio1, 5, 6, 8, 10, 11 : high temperatures in general
	6083005	7.49	43.79	216	
	6088001	7.21	43.65	26	
	6088007	7.28	43.74	238	

* See also PCA graph (Figure SF1)

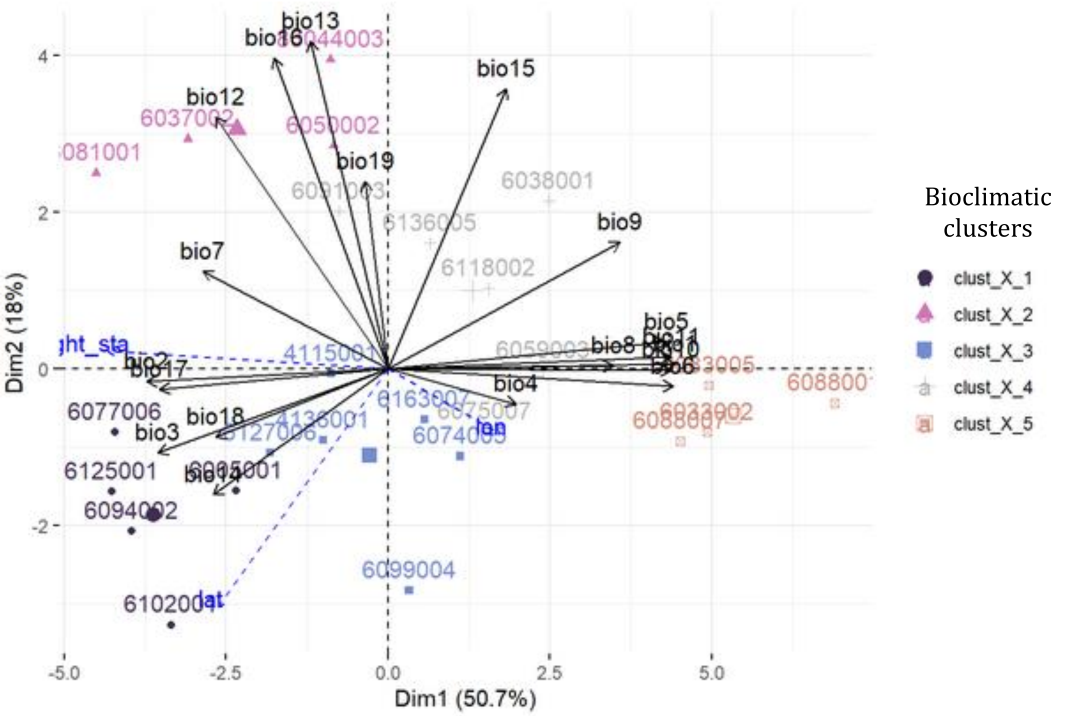

Figure S1. Multivariate analysis of 19 bioclimatic variables from 25 meteorological stations (PCA – 2 main axes), and clustering of the stations from HCPC. Variation on the first axis of the PCA is significantly positively associated with thermic variables (bio1, 6, 11, 10, 5) and significantly negatively correlated with the altitude of the meteorological station. Axis 1 separates cluster 5 from clusters 1 and 2 with clusters 3 and 4 in an intermediate range. The second axis of the PCA is mainly described by precipitation variables (bio13, 16, 15, 12) that differentiate cluster 1 from cluster 2, and cluster 3 from cluster 4.

Table S2. Studied gradients and micro-niche characteristics, structured in groups of variables. Variables in italics were not included in analyses because of redundancy. For biotic micro-niche, contacted plant species and their abundances where combined in four variables, corresponding to the main axes of a Principal Coordinates Analysis (PCoA).

Group of characteristics		Variable	Code name
Ecological gradients and distribution limits		Altitude	Altitude
		Climatic gradient	Clim_grad
		Distance to the centre of *L. pomponium* distribution	Dist_centre
		Distance to the limits of *L. pomponium* distribution	Dist_limit
		Ratio of those distances	*Dist_ratio*
Micro-niche	Topographic micro-niche	Slope	Slope
		Aspect	*aspect*
		Easting (sin(aspect))	easting
		Northing (cos(aspect))	northing
	Soil cover	Lichen cover	Lich_cov
		Moss cover	Moss_cov
		Litter cover	Litt_cov
		Gravel and bare soil cover	GravBSoil
		Block cover	Block_cov
		Stone cover	Stone_cov
		Bedrock cover	BedRock_cov
	Edaphic micro-niche	Water retention capacity	WRC
		Water retention potential	*WRP*
		pH	pH
		Conductivity	conduc
		Organic matter content	*OM*
		Percentage of Carbon	pC
		Percentage of Nitrogen	*pN*
		C:N ratio	*C/N*
	Biotic micro-niche	Number of species	nb_sp
		Shannon diversity	Shannon_div
		Vegetation cover	Vegetation_cov
		1st axis of the PCoA	PCoA-axe1
		2nd axis of the PCoA	PCoA-axe2
		3rd axis of the PCoA	PCoA-axe3
		4th axis of the PCoA	PCoA-axe4

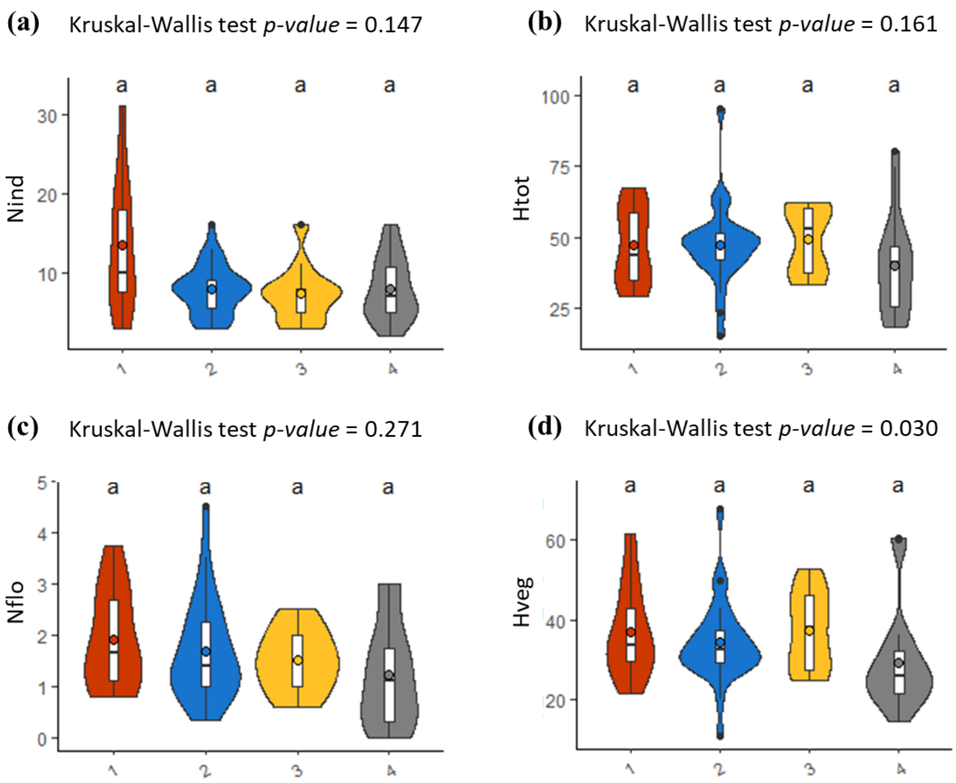

Figure S2. Local abundance and mean phenotype for each quadrat depending on its micro-niche type. Local abundance corresponds to the number of plants in the quadrat (Nind – a), and mean phenotypes are calculated from measurements of four individuals randomly selected in each quadrat. Total height (Htot – b), vegetative height (Hveg – d) and number of flowers (Nflo – c) were measured on the field. Kernel density for each micro-niche type shows quartiles (boxplot) and mean values (coloured circle). Code letters indicate that micro-niche means are significantly ($p < 0.05$) different in pair-wise Wilcoxon tests with Bonferroni adjustment.

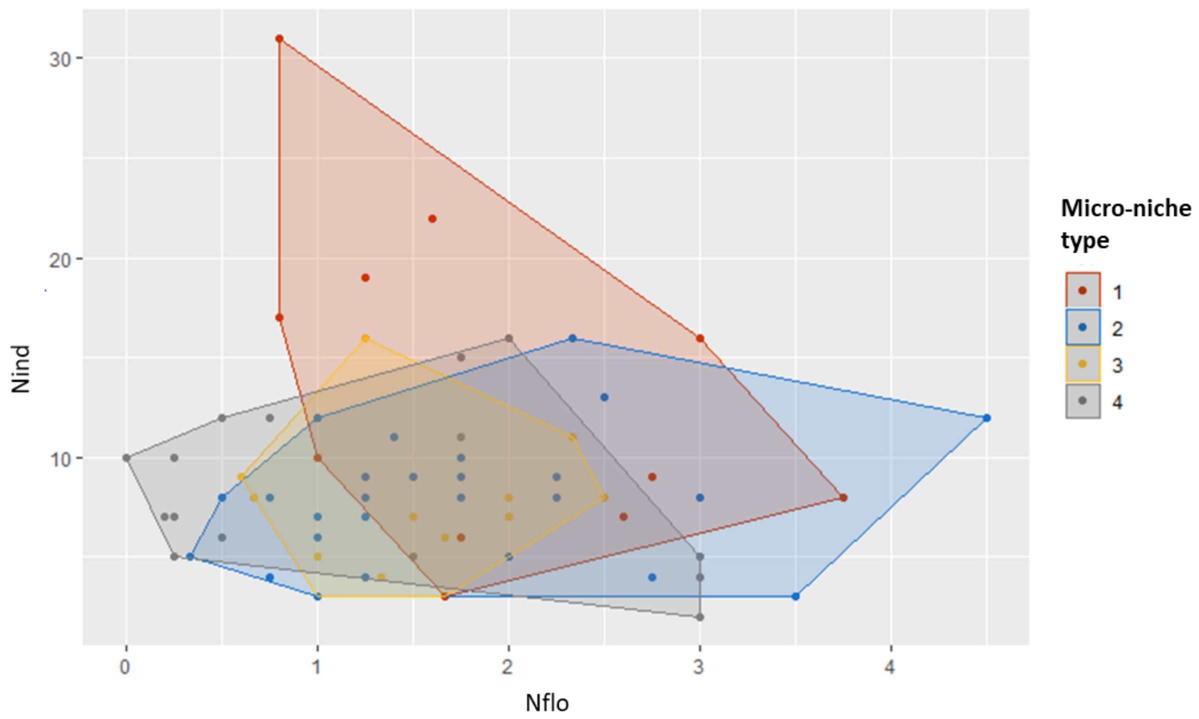

Figure S3. Local abundance and mean number of flowers for each quadrat depending on its micro-niche type. Local abundance corresponds to the number of plants in the quadrat (Nind), and mean number of flowers are calculated from measurements of four individuals randomly selected in each quadrat.

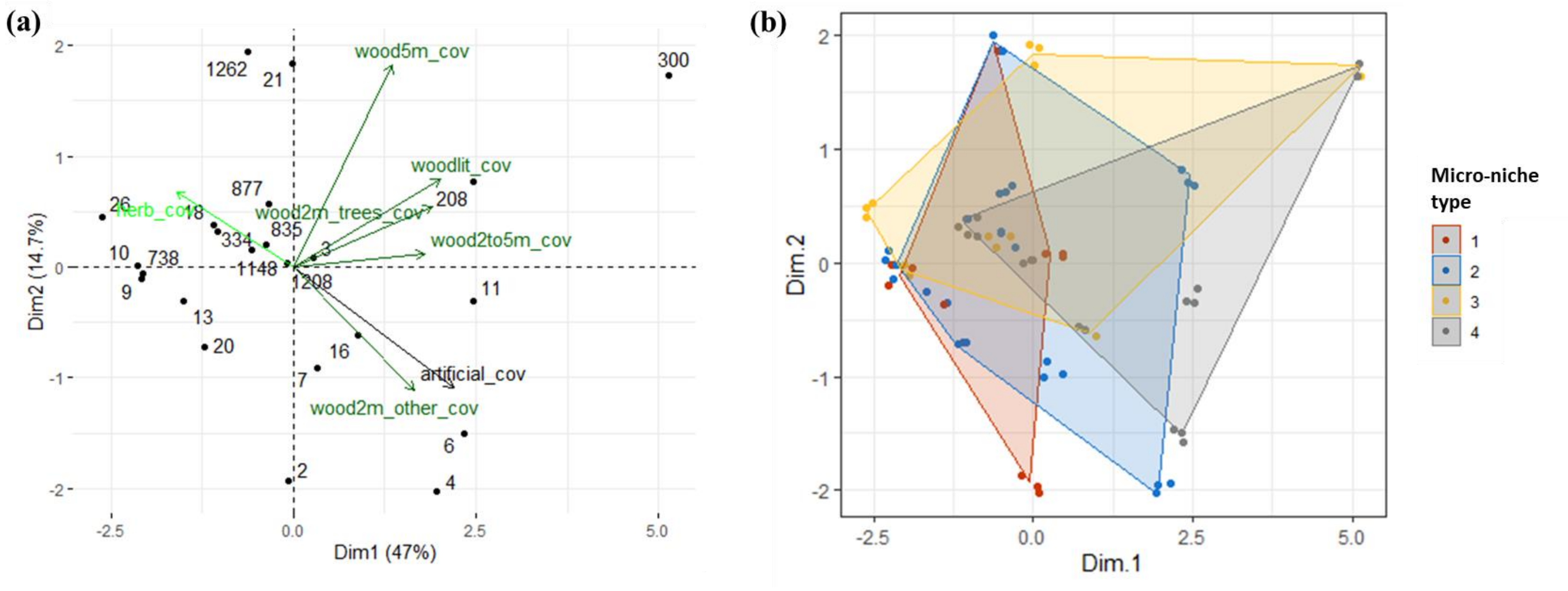

Figure S4. Vegetation structure and artificialization level for sampled locations of *L. pomponium*, evaluated at the site scale (40 data points per site). (a) The biplot of variables and sites from a PCA analysis, variables being coloured by type: woody elements in dark green, herbaceous cover in light green, artificial cover in dark. (b) Representation of the same plan as (a), but sites are divided in their corresponding three quadrats through jittering in order to visualize the repartition of micro-niche types without overplotting.
